# A Proposal to Address NFL Club Doctors’ Conflicts of Interest and to Promote Player Trust

**DOI:** 10.1002/hast.651

**Published:** 2016-11-21

**Authors:** I. Glenn Cohen, Holly Fernandez Lynch, Christopher R. Deubert

## Abstract

*How can we ensure that players in the National Football League receive excellent health care they can trust from providers who are as free from conflicts of interest as realistically possible? NFL players typically receive care from the club's own medical staff. Club doctors are clearly important stakeholders in player health. They diagnose and treat players for a variety of ailments, physical and mental, while making recommendations to the player concerning those ailments. At the same time, club doctors have obligations to the club, namely to inform and advise clubs about the health status of players. While players and clubs share an interest in player health—both of them want players to be healthy so they can play at peak performance—there are several areas where their interests can diverge, and the divergence presents legal and ethical challenges. The current structure forces club doctors to have obligations to two parties—the club and the player—and to make difficult judgments about when one party's interests must yield to another's. None of the three parties involved should prefer this conflicted approach.*

*We propose to resolve the problem of dual loyalty by largely severing the club doctor's ties with the club and refashioning that role into one of singular loyalty to the player‐patient. The main idea is to separate the roles of serving the player and serving the club and replace them with two distinct sets of medical professionals: the* Players' Medical Staff *(with exclusive loyalty to the player) and the* Club Evaluation Doctor *(with exclusive loyalty to the club). We begin by explaining the broad ethical principles that guide us and that help shape our recommendation. We then provide a description of the role of the club doctor in the current system. After explaining the concern about the current NFL player health care structure, we provide a recommendation for improving this structure. We then discuss how the club medical staff fits into the broader microenvironment affecting player health.*

## Article

Football is America's game, but the potential health consequences of the sport are increasingly taking center stage. From major media outlets to federal research funding to conversations among concerned spectators and parents, we are at a moment of unprecedented focus on the potential health consequences, and especially the neurological consequences, of playing football. Concerns about the health of both current and former players in the National Football League have been broad and loud.

There are an estimated 20,000 men alive today who at one time played professional football in the NFL, with thousands more still playing or about to join this elite fraternity. There is an undeniable and urgent need—increasingly recognized by the NFL itself^1^—to advance our understanding of the acute and longer‐term consequences of playing football in order to develop better preventative, diagnostic, and therapeutic interventions. At the same time, it is essential to consider how to minimize the risks for current and future players right now; we should pursue medical research but should not merely await the results. And to truly protect and promote the health of these players, we must address individual factors and structural features simultaneously.

This article focuses on one component of the structural elements affecting NFL player health: the relationship between players and club doctors. How can we ensure that players receive excellent health care they can trust from providers who are as free from conflicts of interest as realistically possible?

NFL players typically receive care from two sets of professionals: club athletic trainers and club doctors, in addition to a variety of other providers unaffiliated with a club, including personal and second‐opinion doctors. Athletic trainers provide the bulk of day‐to‐day care, and they do so under the direction of club doctors. Since trainers and club doctors face similar conflicts of interest in their dual roles of providing services simultaneously to players and to clubs, the discussion that follows is applicable to both, but we focus our analysis on club doctors because of their heightened legal and ethical obligations and their preeminence over the club medical staff.

Club doctors are clearly important stakeholders in player health. They diagnose and treat players for a variety of ailments, physical and mental, while making recommendations to the player concerning those ailments. At the same time, club doctors have obligations to the club, namely to inform and advise clubs about the health status of players. While players and clubs share an interest in player health—both of them want players to be healthy so they can play at peak performance—there are several areas where their interests can diverge, and the divergence presents legal and ethical challenges.

Such problems are not unique to the NFL. They occur in other occupational health settings as well. That said, several elements of the NFL setting (including the limited number of clubs and the seriousness of the involved risks to players) make these problems particularly pernicious. Our proposed solution to the problems might not be applicable in other settings.

The current structure forces club doctors to have obligations to two parties—the club and the player—and to make difficult judgments about when one party's interests must yield to another's. None of the three parties involved should prefer this conflicted approach. We propose to resolve the problem of dual loyalty by largely severing the club doctor's ties with the club and refashioning that role into one of singular loyalty to the player‐patient. The main idea is to separate the roles of serving the player and serving the club and replace them with two distinct sets of medical professionals: the *Players’ Medical Staff* (with exclusive loyalty to the player) and the *Club Evaluation Doctor* (with exclusive loyalty to the club). The Players’ Medical Staff can then serve as an unconflicted and uncompromising champion for player health, while clubs are free to hire additional medical professionals for their distinct needs.

In this article, we begin by explaining the broad ethical principles that guide us and that help shape our recommendation. We then provide a description of the role of the club doctor in the current system. After explaining the concern about the current NFL player health care structure, we provide a recommendation for improving this structure. We then discuss how the club medical staff fits into the broader microenvironment affecting player health, before a brief conclusion.

## Guiding Ethical Principles

In “Protecting and Promoting the Health of NFL Players: Legal and Ethical Analysis and Recommendations,”^2^ of which this article is an outgrowth, we make recommendations for how a wide variety of stakeholders can better protect and promote player health. Although each stakeholder is unique in important ways and may be subject to more specific ethical principles, as we discuss more fully in “Protecting and Promoting the Health of NFL Players,” we derived seven overarching ethical principles to guide our assessment of all stakeholder responsibilities and to structure the nature of our recommendations.

### Respect

The NFL is undeniably a business, but it is a business that relies on individuals who are exposed to substantial risks. These are not passive, inanimate widgets, but persons with inherent dignity and interests, social relationships, and long‐term goals that extend beyond their playing days. No matter how much enjoyment they provide to the half of all Americans who count themselves as professional football fans,^3^ no matter how much revenue they generate, and no matter how much glory comes to players themselves, no stakeholders may treat them “merely as a means” or as a commodity solely for promotion of their own ends.

### Health primacy

Football is a physical game, and injuries ranging from the transient to the severe are relatively common, but these facts do not mean that player health is unimportant any more than they suggest that we may permissibly ignore health risks in other lines of potentially dangerous work. Health is special because it is foundational to all other human pursuits. For this reason, it ought to be accorded special moral weight as compared to other possible goods, and we should be particularly wary in cases where goods will accrue to others whose health is not at risk.

When players are expected or encouraged to sacrifice their health for the game, or even when they are simply not discouraged from doing so, they are potentially treated as mere means. Players have a moral right to have their health protected at the very least, and often they have a right to have it promoted. As a prima facie rule, avoiding serious threats to player health should be given paramount importance in every dealing with every stakeholder. However, there may be instances when a player, acting with full information and with minimal bias or other impairment, may rationally determine for himself that other values—supporting one's teammates, winning, financial rewards, and so on—are more important than his health.

While health matters and indeed is often at the top of any pyramid of human values, we do not maintain that players must, or even should, always choose health over all other goods. This is certainly not a demand that could be made of the general population, and players may be reasonably balancing many different considerations as to what makes a life go well. In some instances, they may reasonably choose to sacrifice their health to some extent. In these cases, we can say that health primacy is giving way to the principle of empowered autonomy (described below). That said, all stakeholders bear an obligation to try to reduce these instances of trade‐off as much as possible and to reject an institution that demands or expects that players sacrifice their health on a regular basis.

**The Football Players Health Study at Harvard University**

In response to concerns about the health of National Football League players, the 2011 collective bargaining agreement between the NFL and the National Football League Players Association included a number of new health, safety, and welfare provisions. One of these provisions sets aside $11 million per year through 2021 to be dedicated to medical research.^1^ In the summer of 2012, the NFLPA issued a request for proposals to conduct original research to be supported by these funds, focusing on “new and innovative ways to protect, treat, and improve the health of NFL players.” The NFLPA's request for proposals specified a number of clinical areas of particular interest, as well as “Medical Ethics (e.g., examination of health care contexts to obtain a better understanding of internal morality of these practices, accountability, new interventions that avoid harms currently incurred, appropriate informed consent in the context of professional athletics, and consideration of medical care in the labor‐management context of professional football).”

After a competitive process, the NFLPA selected to fund Harvard's proposal, including a variety of critical clinical projects alongside a robust set of law and ethics proposals. In February 2014, Harvard Medical School entered into an agreement with the NFLPA to create the Football Players Health Study at Harvard University,^2^ including in its initial phase three main components: a population studies component, which entails research using questionnaires and testing to better understand player health status, wellness, and quality of life, including the largest‐ever cohort study of living former NFL players; a pilot studies program aimed at developing new prevention strategies, diagnostics, and treatments by funding researchers working on innovative and promising developments that have the potential to impact the health of football players; and a Law and Ethics Initiative led by the Petrie‐Flom Center for Health Law Policy, Biotechnology, and Bioethics at Harvard Law School and aimed at understanding the legal and ethical issues that may promote or impede player health and at developing appropriate responsive recommendations.

The Law and Ethics Initiative encompasses a variety of distinct projects, including a comprehensive report entitled “Protecting and Promoting the Health of NFL Players: Legal and Ethical Analysis and Recommendations.” The Law and Ethics Initiative comprises an analysis of the legal and ethical obligations of various stakeholders in NFL player health, a comparative legal and organizational policy analysis of various professional sports leagues to identify best policies in protecting player health, a qualitative interview study with NFL players and their families to better understand their legal and ethical concerns related to health, and an analysis of the legal and ethical implications of current and potential medical tests and devices that might be used by NFL clubs and players.

“Protecting and Promoting the Health of NFL Players” is based on a belief that protecting and promoting the health of professional football players, and by extension athletes of all ages and types, requires addressing *individual* factors and *structural* features simultaneously. In other words, we need an integrated systems approach at the level of the player‐patient and his circumstances.

The report also takes a broad view of what health is and of what influences it. Clinical conditions and physical symptoms are primary components of health, but a focus on only these elements misses something crucial. Health is not merely the absence of physical or mental malady, the freedom from disease, but, rather, the attainment and maintenance of excellent mental and bodily vigor.^3^ Any attempt to segregate the clinical and nonclinical aspects of health will miss the important—and potentially causal—interrelationships between the two. Along these lines, there has been a trend over several decades to acknowledge the social determinants of health. These go beyond the sorts of things for which one would seek out a doctor's care and include broadly “the conditions in which people are born, grow, live, work, and age,” as affected by the “distribution of money, power, and resources at global, national and local levels.”^4^

This article is an outgrowth of “Protecting and Promoting the Health of NFL Players”; it, too, is informed by the integrated systems approach discussed above. However, the full report focuses on a wide range of stakeholders and issues, while here we focus on one set of stakeholders and one structural element: club doctors and their inherent conflict of interest given their dual role of providing medical care to players and strategic advice to clubs.

Recognizing that this problem is deeply entrenched and that the recommendation we develop is a significant departure from the status quo, we invited a diverse and highly qualified group of experts to comment on this article. In addition to the individuals and organizations whose commentaries appear in this special report, we invited a Hall of Fame former player and Betsy Nabel, the NFL's chief health and medical advisor and the president of Brigham and Women's Hospital in Boston, to provide commentaries. Both respectfully declined, citing time constraints. We greatly appreciate the engagement and consideration of each commentator.

1. National Football League and National Football Players Association, “Collective Bargaining Agreement,” August 4, 2011, art. 12, § 5.
2. The contract governing this project protects our academic integrity as researchers; it does not give any external party any editorial control over our work. A version of our report, “Protecting and Promoting the Health of NFL Players: Legal and Ethical Analysis and Recommendations,” from which this article is derived, was shared with the NFLPA, NFL, and other stakeholders prior to publication. The NFLPA was treated the same as other stakeholders, with the exception of a contractually guaranteed thirty‐day review to ensure that we did not use any confidential information. We considered all feedback provided to us but retained final editorial control. The content is solely the responsibility of the authors and does not necessarily represent the official views of the NFLPA or Harvard University. We deemed these safeguards essential to protecting our academic integrity but nonetheless recognize that there are inherent conflicts of interest in funded research. See Institute of Medicine, Board on Health Sciences Policy, “Conflict of Interest in Medical Research, Education, and Practice,” 2009, http://www.nationalacademies.org/hmd/Reports/2009/Conflict‐of‐Interest‐in‐Medical‐Research‐Education‐and‐Practice.aspx, and D. F. Thompson, “Understanding Financial Conflicts of Interest,” New England Journal of Medicine 329 (1993): 573‐76. The Football Players Health Study supports 20 percent of Cohen's, 30 percent of Lynch's, and 100 percent of Deubert's salary. From August 2010 to May 2014, Deubert was an associate at the law firm of Peter R. Ginsberg Law, LLC (formerly known as Ginsberg & Burgos, PLLC). During the course of his practice at that firm, Deubert was involved in several legal matters in which the NFL was an opposing party.
3. See World Health Organization, preamble to the Constitution of the World Health Organization as adopted by the International Health Conference, New York, 19‐22 June, 1946, signed July 22, 1946, by the representatives of sixty‐one states (Official Records of the World Health Organization, no. 2, p. 100) and entered into force on April 7, 1948, at http://www.who.int/about/definition/en/print.html.
4. World Health Organization, “Social Determinants of Health,” http://www.who.int/social_determinants/sdh_definition/en/; see also M. Marmot and R. G. Wilkinson, eds., Social Determinants of Health: The Solid Facts, 2nd ed. (Copenhagen, Denmark: World Health Organization, 2005).

### Empowered autonomy

Serious risks to health in football must be minimized as a structural matter. Beyond that, though, players are ultimately the ones who are most able to make decisions and take steps to protect and promote their health. In order to effectively do so, however—like all patients—they often need support and empowerment. They need trustworthy factual information presented in a readily understandable way, as well as decision‐making tools that help them see not only short‐term benefits and costs but also longer‐term implications. They need to have unfettered access to competent doctors, contract advisors (that is, agents), financial advisors, and others they trust. The goal is not merely to allow players to choose for themselves which capabilities and values to prioritize, but to promote informed and authentic choice. Such choice also requires that players have access to good options and alternatives—unconflicted and qualified medical advisors, educational opportunities and assistance with postplay career transitions, and the like—with the freedom to select among them without undue pressure from others.

Although it may not be a perfect resolution of the various background pressures players may face, ensuring that player choice regarding matters related to their health will be free from misinformation, lack of understanding, bias, and avoidable negative influences is essential. Other stakeholders have a responsibility to help achieve these criteria whenever possible. Where these criteria are lacking, such as when a player is cognitively impaired or has unresolved biases, the principle of health primacy reigns supreme.

### Transparency

Again, to avoid treating players as mere means and to promote players’ empowered autonomy, all parties should be transparent about their interests, goals, and potential conflicts as they relate to player health. Failure to be so disrespects players and may also result in the inappropriate subrogation of player health to other interests. Thus, players should immediately receive information relevant to their health—that is, medical information specific to them individually, information about risks to players in general (including emerging information that would be sufficiently credible to be taken seriously by experts even if it is not fully validated), and information about relationships that could influence judgment and recommendations related to player health. Promoting transparency will allow players to make better decisions for themselves and will promote trust in all those who play a role in their health.

### Management of conflicts of interest

While it is helpful to explain to players where conflicts of interest exist, since this may allow them to be on guard to better protect their own interests, mere disclosure will not help players when sufficient alternatives are lacking. Instead, all stakeholders should take steps to minimize conflicts of interest and to manage them when they cannot be eliminated. Often conflicts of interest are painted as nefarious or as the result of bad intentions by bad actors, but that is not necessarily the case. Many conflicts of interest are structural—the way in which a system is set up makes it hard for even well‐intentioned and ethical individuals to do the right thing. When structure is the problem, it is structure that must be changed. Among other things, this will often involve removing problematic incentives, altering conflicted relationships, and creating separate and independent sources of advice. In the case of the NFL, it will also entail auditing the behavior of those with incentives that diverge from the primacy of player health. This principle is particularly important for the focus of this article, the relationship of players and the club medical staff.

### Collaboration and engagement

As we discuss in greater depth below, protecting and promoting the health of professional football players depends on many parties who should strive to act together whenever possible to advance that primary goal. Players should be engaged by stakeholders in all matters that influence their health.

### Justice

Finally, all stakeholders have an obligation to ensure that players are not bearing an inappropriate share of risks and burdens compared to benefits reaped by other stakeholders. Stakeholders should also be aware of the ways in which changing rules, laws, or programs—such as trading benefits to former players for benefits to current players—may have differential effects on certain subcategories of players, and they should be attuned to ways in which those disadvantages can be blunted or recompensed. The principle of justice also demands awareness of how practices can have implications beyond the NFL. The way in which player health is protected and promoted at the top echelons of the sport will influence policies, practices, and culture all the way down the line, influencing the health not only of future NFL players but also the vastly larger pool of Americans who will play football and never make it to the NFL.

## Club Doctors in the Current System

### Who are they?

The collective bargaining agreement reached in 2011 between the NFL and the NFL Players Association—the key document that governs the relationship between and among players, clubs, the NFL, and the NFLPA—requires that each club “retain” a board‐certified orthopedic surgeon and at least one physician board certified in internal medicine, family medicine, or emergency medicine.^4^ All physicians must also have a certificate of added qualification in sports medicine (or be grandfathered in).^5^ In addition, clubs are required to retain consultants in the neurological, cardiovascular, nutritional, and, neuropsychological fields.^6^ While each club generally has a single “head” club doctor, about 175 doctors work with NFL clubs in total^7^—an average of 5.5 per club. Most if not all of the doctors retained by NFL clubs are members of the National Football League Physicians Society, the professional organization for club doctors.

Club doctors are chosen by and report to the club's executives.^8^ They are affiliated with a wide variety of private practice groups, hospitals, academic institutions, and other professional sports leagues; some of these institutions have long‐standing relationships with clubs that often influence clubs’ decisions to retain doctors. The NFLPA currently plays no role in the selection of club doctors other than to ensure that they have the required qualifications and credentials.

Club doctors are one component of the more expansive club medical staff. Various medical professionals provide health care to players, including but not limited to athletic trainers, physical therapists, massage therapists, chiropractors, dentists, nutritionists, and psychologists. Club doctors and athletic trainers have the most systematic and continuous relationships with players as compared to these other professionals, and they are generally the principal health care providers for the players. However, athletic trainers, acting under the direction of the club doctors, are also an integral part of player care.

### What do they do?

At the outset, it is important to stress the source of the structural conflict of interest: club doctors provide care to players while also having some type of contractual or employment relationship with, and thus obligations to, the club. A club doctor's principal responsibilities are (1) providing health care to players, (2) helping players determine when they are ready to return to play, (3) helping clubs determine when players are ready to return to play, (4) examining players the club is considering employing, and (5) helping clubs to determine whether a player's contract should be terminated because of the player's physical condition (for example, deciding whether an injury will prevent the player from playing again).^9^ The first two responsibilities might be considered “services to players”; here, the club doctor is treating and advising the player, including taking into consideration the player's athletic and other goals. The last three responsibilities might be considered “services to the club”; here, the doctor is exclusively advising the club. Nevertheless, the club doctor's two roles are not and cannot be separated in practice. It is the club doctors’ simultaneous obligations to both players and the club that present the conflict of interest concerns that are the focus of this article. This inherent conflict exists throughout the NFL, although the practices and experiences of club doctors may vary somewhat from club to club. For example, some clubs may be more actively engaged with club doctors, while others may be more hands‐off.

During the regular season, club doctors generally visit the club for a few hours twice a week to address player injuries, and they are also present on game days.^10^ Club doctors are in regular communication with the club's athletic trainers about player status,^11^ and they rely on the athletic trainers to monitor and handle the players’ care during the week.^12^ All club athletic trainers work under the supervision of a club doctor, and in fact, state licensing statutes and regulations require athletic trainers to work under the direction of a licensed physician.^13^ Nevertheless, athletic trainers are often the first and most consistent source of medical care provided to players. During the week, athletic trainers are responsible for treating ongoing injuries.

On game days, a variety of medical professionals are involved in player care. Each club generally has four athletic trainers, two orthopedists, two primary care physicians, and one chiropractor present.^14^ In addition, pursuant to the NFL Head, Neck and Spine Committee's Protocols Regarding Diagnosis and Management of Concussion (the “concussion protocol”), which dictates how the clubs treat players who have suffered or are suspected of having suffered a concussion, each club is designated an unaffiliated neurotrauma consultant to assess possible concussions.^15^ A variety of other medical professionals are also available to both clubs, including an independent athletic trainer who views the game from the press box to spot possible injuries (the “spotter”),^16^ an ophthalmologist, a dentist, a radiology technician to handle the stadium's x‐ray machine, an airway management physician, and an emergency medical technician or paramedic crew. In total, twenty‐seven medical personnel members are typically on hand at an NFL game.^17^

The club medical staff is responsible for keeping the club apprised of players’ medical conditions. Players execute waivers (which are collectively bargained with the NFLPA) permitting the club doctors and athletic trainers to disclose players’ medical information to club employees, such as coaches and the general manager. As club doctors have part‐time relationships with the clubs, the responsibility generally falls on athletic trainers to keep coaches and general managers apprised of players’ injury statuses during regular meetings to enable the general manager to decide whether to sign another player in the event a player is unable to play.

The relationship between club medical staff and players is obviously important. A 2016 Associated Press survey of one hundred current NFL players posed the question whether “NFL teams, coaches and team doctors have players’ best interests in mind when it comes to injuries and player health.”^18^ Forty‐seven players answered yes, thirty‐nine answered no, and fourteen were either unsure or refused to respond.^19^

We also spoke with several former and current players to get a better understanding about NFL player health issues.^20^ These interviews were intended to be illustrative but not necessarily representative of all players’ views, and they should be read with that limitation in mind. Most of the players we spoke to said that the current structure of the club medical staff generated distrust of club doctors, but this feeling was not universal:

- *Current Player 1:* “I do trust our team doctors. Any time that I've dealt with them, they've been very upfront with me and gave me all the information I needed about my injuries. I never got the impression that they were hiding anything from me or putting me into a dangerous situation.”^21^
- *Current Player 2:* “I certainly think that there are a number of players that do not trust club doctors, and for various reasons. They feel as though those doctors work for the team and they do what's in the best interests of (a) the coach and (b) ownership. And I think that a lot of times players feel as though these doctors maybe don't disclose the full extent of their injuries [and] give them a hard time about getting second opinions.”
- *Current Player 3:* “I think that there are some instances where they don't trust the team doctors because they don't like the team, and the team doctor just wants them to get back on the field …. I think sometimes the doctors may … not tell you the full extent of what's going on … about a certain injury. [But] I think there [are] sometimes team doctors where the players trust them and the doctors are great and very trustworthy.”
- *Current Player 4:* “I do not trust team doctors. I've had multiple occasions where I've had a team doctor tell me one thing and then I go and have a second opinion and I get a completely different answer …. [T]he club doctor has the same mentality as the club itself. More than anything, they want a player on the field…. I feel like the team doctor only has the best interest of the team in mind and not necessarily the player.”
- *Current Player 5:* “My trust level with [my former club doctor] was very high. I know a lot of guys respected him. But I know there was a number of guys that had disagreements with him…. But I think generally the guys that have a problem with the doctors are guys that have had some sort of injury that affects their career and their ability to make money and support themselves and their families.”
- *Current Player 7:* “[T]hey're doing and saying what's best to get you back on the field as soon as possible.”
- *Current Player 8:* “I don't feel like they are diagnosing or at least treating us like they would want to be treated or how they would treat their kids …. [T]hey're going to lean towards what keeps you on the field.”
- *Current Player 9:* “I've seen times when the medical staff has lied about injuries.”
- *Current Player 10:* “I've always had good relationships and good positive vibes from the doctors that have been out on the field…. I think players trust them; I think the agents don't.”
- *Former Player 2:* “[T]hese doctors are good. I wouldn't say they are great. You know, at the end of the day … the organizations are paying the doctors…. I would say probably 65 percent of the team trusts the doctor, and probably 35 percent of the team does not.”
- *Former Player 3:* “My experience has always been very positive…. I know that players are told, or maybe just a little bit skeptical or suspicious of docs, thinking that they have the team's interest in mind first before the player's, but I never had an experience where I thought that was the case.”

Players we interviewed also indicated that the communications between the club medical staff and the coaches and general manager put pressure on players to practice and cause them to withhold information from the medical staff.^22^ Current Player 3 expressed this view: “Sometimes they want you out there and they want to see if you can push through certain pain if the doctor feels like, okay, it's not going to get any worse if you play. You just have to deal with the pain. Can you push through that pain? I think sometimes they want to see those types of things.” Players often do not want to tell the medical staff that they are not healthy enough to practice for fear that the medical staff will relay that message to the general manager, with the suggestion that the general manager consider signing a potential replacement player. Current Player 8 said, “I go into those meetings [with the athletic trainer] very conscious of the fact that anything I say or do, it's going to be relayed to the people who are there to determine my future.”

To be sure, not all players share this view of the relationship between players and club medical staff, and the relationship can differ from club to club and over time. St. Louis Rams club doctor and former president of the NFLPS Matthew Matava maintains that the club's on‐field success bears no relation to the club doctor's obligations or status with the club:

Physician jobs are not dependent on wins and losses…. I've survived 1‐15, 2‐14 and 3‐13 seasons with the Rams. We can go 0‐16, and my job does not change one iota…. Obviously we know that we want to have the guys back on the field as quickly as they can be in a safe fashion—and we can be creative in the ways we do so—but there are no competitive issues involved in our decision to return to play.^23^

At the conclusion of the season, the club medical staff performs end‐of‐the‐season physicals on the players. While the physicals can benefit the players by revealing injuries or conditions in need of care, they also provide important benefits to the club. The physicals can provide the club with a record that at the end of the season the player was healthy, so that if the player's contract is terminated during the off‐season, the player cannot claim that his termination was due to injury and try to obtain compensation through an injury grievance or the injury protection benefit.^24^ The club can also use the assessment of each player's health in deciding whether to retain the player for next season.

Finally, club medical staff has an additional important role in advising the club on which rookie players to draft. Each off‐season, members of clubs’ medical staffs attend the NFL Scouting Combine, an annual event held each February in which approximately three hundred of the best college football players undergo medical examinations, intelligence tests, interviews, and multiple football and other athletic drills and tests.^25^ NFL club executives, coaches, scouts, doctors, and athletic trainers attend the Combine to evaluate players for the upcoming NFL draft, which is usually in April.^26^ According to the NFLPS, the role of the club doctor at the Combine “is to obtain a comprehensive medical and orthopaedic assessment of every player that is going to be part of the NFL Draft.”^27^ Also according to the NFLPS, “[T]he team physicians along with their athletic training staff assess every player who is going to be available for the NFL Draft and provide a report back to the scouting department, the head coach, the general manager and the front office about the medical condition of each player. This information becomes very important in a team's assessment of whether or not a player will be drafted.”^28^ Club doctors do not provide medical care to any player at the Combine.

## Club Doctors’ Dual Obligations

Club doctors are clearly vitally important for protecting and promoting player health. Yet given the various roles just described, they face an inherent structural conflict of interest. Recognizing this conflict does not lead to any moral judgment about them as competent professionals or devoted individuals; the conflict is simply a fact of the current organizational structure, in which club doctors simultaneously perform two roles that are not necessarily compatible with each other. On the one hand, they are hired by clubs to provide and supervise player medical care. As a result, they enter into a doctor‐patient relationship with the players and have a legal and ethical responsibility to protect and promote the health of their player‐patients, in line with players’ interests as defined by the players themselves. This means providing care and medical advice aligned with player goals and working with players to help them make decisions about their own self‐protection, including when they should play, rest, and retire.

On the other hand, clubs engage doctors because medical information about and assessment of players is necessary for the clubs’ decisions about a player's ability to perform at a sufficiently high level in the short‐ and long‐term. These are business decisions. Additionally, clubs engage doctors to advance the clubs’ interest in keeping their players healthy and helping them recover as fully and quickly as possible when they are injured. These dual roles can conflict with each other because players and clubs themselves can have conflicting interests. Yet club doctors are called on to serve both parties.

Club doctors are not alone in facing these conflicts. Many doctors provide care to employees in a variety of occupational settings, such as in the military, law enforcement, and factories and other industrial settings,^29^ and in these settings as well, doctors can be conflicted between doing what is best for the employee and what is best for the employer. Our review of the legal and ethical literature on occupational medicine did not reveal any clear resolution or guidance with bearing on the context of professional sports medicine, however.^30^ Thus, we have crafted an approach that we believe meets the specific context of the NFL.

While the practical impact of these conflicts almost certainly varies from club to club depending on the club's approach to player health and the medical staff's autonomy, the conflict itself is unavoidable as long as the club doctor is expected to wear both hats. Some doctors may be able to negotiate the conflict better than others, but in general, a system that requires heroic moral and professional judgment in the face of a systemic structural conflict of interest is one that is bound to fail. Moreover, even if a club doctor can manage the conflicts, their mere existence can compromise player trust, which is a critical element of the doctor‐patient relationship. This is what it means for the conflict to be inherent; the conflict is rooted in the perceptions of others as much as in the decisions and actions of the conflicted party. It is the *system* that deserves blame, not individual doctors.

Existing regulatory, ethical, and legal standards provide some guidance on this issue. However, the standards can be contradictory, lack clarity, or otherwise fail to resolve these concerns.

### The approach in the collective bargaining agreement

The collective bargaining agreement between the NFL and the NFLPA contains a provision governing the club doctor's standard of care:

[E]ach Club physician's primary duty in providing medical care shall be not to the Club but instead to the player‐patient. This duty shall include traditional physician/patient confidentiality requirements. In addition, all Club physicians and medical personnel shall comply with all federal, state, and local requirements, including all ethical rules and standards established by any applicable government and/or other authority that regulates or governs the medical profession in the Club's city.^31^

This CBA provision is susceptible to multiple interpretations. On a generous reading (that is, one that does not give the words “in providing medical care” any special emphasis), club doctors’ primary duty is to the player at all times. On a less generous reading, the CBA provision demands a primary duty to the player‐patient *only* when the club doctor is “providing medical care,” and it is inapplicable when the club doctor is rendering services to the club. However, given how club doctors are currently situated within the club, the two roles assigned to them cannot be truly separated, and their duties cannot possibly be exclusively to the players. Providing care to the player occurs simultaneously with performing duties for the club by judging the player's ability to play and help the club win.

Thus, the club doctor is required by the CBA to provide medical care that puts the player‐patient's interests above the club's (in the event that these interests conflict). This is as it should be. However, in most instances—and as seemingly recognized by the CBA—it is impossible under the current structure for the club doctor to always have a primary duty to the player‐patient over the club because sometimes the club doctor is not providing care but, rather, is advising the club on business decisions. In other words, the club doctor cannot always hold the player's interests as paramount and at the same time abide by his or her obligations to the club.^32^ Indeed, a club doctor could provide impeccable player‐driven medical care (treating the player‐patient as primary, in accordance with the CBA) while simultaneously hurting a player's interests by advising the club that the player's injury will limit his ability to help the club. Thus, under any reading of the CBA provision, players lack a doctor who is concerned only with their best interests at all times.

The CBA provision also seems to require that the care relationship between players and club doctors be afforded traditional confidentiality protections. However, the players we interviewed indicated that, in practice, clubs as a matter of course request or require players to execute statements effectively waiving this requirement—and that no player refuses to sign the waiver. (Current Player 5 called attention to this: “[O]ur first day back in camp, we sign a ton of stuff. I believe one of them is [a] medical release form that allows our team doctors to discuss medical conditions with team officials …. I've seen some guys question some of the documents we have to sign, but when you're given a stack of papers and it's you sign this and you play football or you don't sign it and you don't, everybody signs it. I don't know anybody who hasn't.”) The waivers (which are collectively bargained with the NFLPA) permit the athletic trainer and club doctors to disclose the player's medical information to club employees, such as coaches and the general manager. Thus, it is unclear what work the confidentiality protections in the CBA are doing. Of course, if clubs are receiving medical information about the players, players will be less than forthcoming about their medical needs.

### The relevant ethical standards

In examining and considering solutions to minimize this conflict of interest in the triad between players, club doctors, and clubs, we reviewed several codes of ethics relevant to doctors. As noted above, NFL clubs retain a wide range of doctors, including orthopedists, internists, family medicine specialists, emergency medicine specialists, neurologists, neurosurgeons, cardiologists, and psychologists, among others.^33^ Each of the specialties generally has its own professional societies and organizations, which might also have ethical codes or practice guidelines relevant to the specialty. Similarly, there are also codes of ethics specific to doctors working in occupational settings. For example, the American College of Occupational and Environmental Medicine has a code of ethics,^34^ as does the International Commission on Occupational Health.^35^ These documents provide important direction on appropriate and best practices. The most prominent code of ethics for doctors, however, is the American Medical Association's *Code of Medical Ethics*.^36^ Since the AMA is a voluntary organization, its code does not itself have legal force. However, many state licensing boards have codes of ethics that reference or are substantially similar to the AMA's,^37^ and violations of these boards’ codes of ethics may result in disciplinary actions such as revoking a physician's license to practice medicine.^38^ Given the importance of the AMA code, we focus our discussion on it.^39^ We also rely on a code of ethics published by the Féderation Internationale de Médicine du Sport, the leading international sports medicine organization.^40^ The FIMS code is significant because it is specific to sports. As the NFLPS does not have a code of ethics—in “Protecting and Promoting the Health of NFL Players,” we recommend they adopt one—there is nothing specific to football upon which to draw.^41^

The AMA code clearly acknowledges the issue at hand:

Physicians who are employed by businesses or insurance companies, or who provide medical examinations within their realm of specialty as independent contractors, to assess individuals’ health or disability face a conflict of duties. They have responsibilities both to the patient and to the employer or third party.^42^

The AMA code also provides more specific guidance. In situations where the doctor is providing treatment to a patient, the AMA code is clear that the doctor's principal obligation must always be to the patient. The AMA code's first principle is that “[a] physician shall be dedicated to providing competent medical care, with compassion and respect for human dignity and rights.”^43^ Similarly, the AMA code's eighth principle declares that “[a] physician shall, while caring for a patient, regard responsibility to that patient as paramount.”^44^ Additionally, the code dictates that “[t]he relationship between a patient and physician is based on trust, which gives rise to physicians’ ethical responsibility to place patients’ welfare above the physician's own self‐interests or obligations to others, to [use] sound medical judgment on patients’ behalf, and to advocate for their patients’ welfare.”^45^ Finally, it dictates that “[w]here the economic interests of the hospital, health care organization, or other entity are in conflict with patient welfare, patient welfare takes priority.”^46^ These provisions mirror the CBA language described above, but it is important to recognize that many doctors in ordinary practice do not have such stark dual obligations as those bearing on club doctors; focusing on their responsibilities only while providing treatment is not as problematic for them as it is for doctors providing treatment while being employed by an NFL club.

The AMA code contains a sports‐specific provision requiring doctors to put the athlete's interests ahead of their own or anyone else's.^47^ It also contains guidance for doctors who might be employed or supervised by nonphysicians (as may be the case for club doctors): “Physicians who are … employees of nonphysician practitioners must: (a) [g]ive precedence to their ethical obligation to act in the patient's best interest [and] (b) [e]xercise independent professional judgment, even if that puts the physician at odds with the employer/supervisee.”^48^

The FIMS code of ethics contains considerable guidance for club doctors concerning conflicts of interest, including that “[t]he physician's duty to the athlete must be his/her first concern and contractual and other responsibilities are of secondary importance,”^49^ that “[a]dvice given and action taken should always be in the athlete's best interest,”^50^ and that club doctors “must insist on professional autonomy and responsibility for all medical decisions concerning the health, safety and legitimate interest of the athlete. No third party should influence these decisions.”^51^ Moreover, “[i]t is the responsibility of the sports medicine physician to determine whether the injured athletes should continue training or participate in competition. The outcome of the competition or the coaches should not influence the decision, but solely the possible risks and consequences to the health of the athlete.”^52^

These provisions might suggest that the issue is resolved under existing ethical codes: that club doctors have an unwavering obligation to the patient. However, these ethical obligations do not account for all of a club doctor's obligations. When club doctors provide care and treatment to players, they are engaged in a doctor‐patient relationship.^53^ At the same time, however, clubs engage doctors because medical information about and assessment of players is necessary to clubs’ decisions related to a player's ability to perform at a sufficiently high level. When the club doctor is providing services to the club, such as examining a player for purposes of advising the club, there may be only a limited doctor‐patient relationship (or none at all), which creates different legal and ethical obligations. What this means is that club doctors may find themselves to have two different types of relationships with player‐patients, depending on the circumstances, with different and conflicting obligations.

To understand these different obligations, consider AMA code opinion 1.2.6, in contrast with the ethical parameters set forth above. Opinion 1.2.6 states that “industry‐employed physicians or independent medical examiners establish *limited* patient‐physician relationships. Their relationships with patients are limited to the isolated examination”^54^ (emphasis added). There are at least three kinds of interactions between players and club doctors that can be usefully juxtaposed against this opinion.

At one end of the spectrum, there is no treatment relationship at all. For example, club doctors might examine dozens of players at the NFL Combine, only a few of whom will ever actually join the club and be treated by the club doctor. In these situations, opinion 1.2.6 lends useful guidance. When there is only a limited relationship, the doctor has the following obligations:

(a) Disclose the nature of the relationship with the employer or third party and that the physician is acting as an agent of the employer or third party before gathering health information from the patient.
(b) Explain that the physician's role in this context is to assess the patient's health or disability independently and objectively. The physician should further explain the differences between this practice and the traditional fiduciary role of a physician.
(c) Protect patients’ personal health information in keeping with professional standards of confidentiality.
(d) Inform the patient about important incidental findings the physician discovers during the examination. When appropriate, the physician should suggest the patient seek care from a qualified physician and, if requested, provide reasonable assistance in securing follow‐up care.^55^ [*sic.*]

The doctor has no obligation to monitor or treat the individual after the examination.^56^

In the middle of the spectrum are situations in which the scope of a doctor‐patient relationship is not clear. For example, when club doctors perform preseason physicals on players, no treatment relationship has yet been established, although arguably that physical is the beginning of a doctor‐patient relationship rather than an isolated assessment.

At the other end of the spectrum is the most common factual scenario: the frequent and in‐depth treatment encounters between players and club doctors. These encounters are generally not isolated but are instead ongoing and involve care and treatment rather than just examination. This is the most typical scenario in which club doctors interact with players, and it is clear that the AMA code opinion 1.2.6 does not apply in these settings.

In this, the most typical setting, the current structure requires the club doctor to do the impossible: to meet the ethical responsibilities that apply when providing services to a patient and those that apply when providing services to an employer. Club doctors cannot simultaneously have both *complete* and *limited* doctor‐patient relationships with players because these roles demand the doctor do incompatible things. If they are deemed to have traditional doctor‐patient relationships, then their ability to satisfy obligations to the clubs is dramatically limited; and the opposite is true if they are deemed to have only limited relationships with players. Club doctors cannot simply go back and forth between their two roles and the concomitant responsibilities because the responsibilities conflict. Thus, these existing ethical standards ultimately fail to resolve the structural conflict of interest inherent to the NFL workplace (and to many other occupational health settings).

Beyond treatment alone, we see an additional problem regarding the club doctor's specific obligations concerning confidentiality of player medical information. The AMA code suggests that club doctors engaged in typical doctor‐patient relationships must steadfastly protect the confidentiality of player information.^57^ However, it also recognizes that an employee can sometimes authorize the disclosure of his or her medical information to an employer.^58^ Similarly, the FIMS “Code of Ethics” also declares that “[i]t is essential that each athlete … authorizes disclosure of otherwise confidential medical information, but solely to the specific responsible persons and for the expressed purpose of determining the fitness of the athlete for participation.”^59^ Thus, NFL clubs’ practice of requiring players to authorize disclosure of their medical information in the employment context seemingly comports with existing ethical codes.^60^

### Relevant law

The current structure also complicates club doctors’ legal obligations. A doctor has a *legal* obligation to act in the best interests of the patient at all times that there is a doctor‐patient relationship, just as he or she has an *ethical* obligation to do so.^61^ However, club doctors’ obligations become less clear when factoring in services to the club, such as examining players and advising the club about a player's health. This is because courts have generally held that doctors performing medical examinations for nontreatment purposes have only a limited patient‐physician relationship, with the limited duty of exercising care consistent with their professional training and expertise so as not to cause physical harm by negligently conducting the examination.^62^ However, in the legal cases in which this issue has arisen, the doctors performing the medical examinations did not also have a simultaneous treatment relationship with the patient. Thus, the court opinions—like the ethical codes—do not address or adequately encompass the complexities of the club doctor‐player relationship and whether a club doctor could alternate between having comprehensive and limited obligations to patients depending on the particular role being performed.

NFL clubs’ practices concerning confidentiality of player medical information seem to comport with the relevant law, which is weaker than provisions governing conflicts of interest. As a general rule, assuming a doctor‐patient relationship exists, doctors have both common law and statutory obligations to keep patient information confidential.^63^ That said, a number of exceptions are likely applicable to information shared between players and club doctors. To start, the federal Health Insurance Portability and Accountability Act probably governs club doctors’ requirements concerning the confidentiality of player medical information.^64^ However, the waivers executed by players probably provide the authorization required by HIPAA, and even without the authorizations, club doctors are probably permitted by HIPAA exceptions to provide certain health information about players to the clubs.^65^ Furthermore, twenty‐two states in which NFL clubs play or practice (all except Wisconsin) have statutes that permit health care providers to provide employers with an employee's medical records and information in some situations,^66^ such as when employers are assessing potential or actual workers’ compensation claims and procuring payment for their services. Finally, a doctor‐patient relationship is required for a doctor to be subject to common law and statutory confidentiality requirements,^67^ and as noted above, in some contexts, there may be only a limited doctor‐patient relationship between club doctors and players.

## Toward Restructuring the Club Medical Staff

The existing ethical codes and legal requirements are not adequate to ensure that players receive health care they can trust from providers who are as free from structural conflicts of interest as is realistically possible. Of course, achieving this goal is legally, ethically, financially, and structurally complicated.

Given the ethics of the doctor‐patient relationship, it is clear that club doctors must never sacrifice player health in order to advance club interests. They must not, for example, for the sake of the team recommend treatment that will get a player back on the field quickly but substantially harm the player's health. This is not to say that clubs do not have a legitimate interest in player health and player health information. Because player health significantly affects clubs’ ability to win and so affects their business, the clubs must have access to some information about player health and medical treatment, including sufficient information to assess whether a player should play. Similarly, clubs have a legitimate interest in understanding players’ short‐ and long‐term health prospects so that they can make informed decisions about the players’ short‐ and long‐term prospects of assisting the club. This is the stark reality of a business driven by physical prowess and ability, but we believe there are preferable mechanisms to acknowledge that reality while accounting for player interests than are offered by the existing system.

Since the CBA takes the dual roles of club doctors as a given, it cannot fully advance player health as a club doctor's primary concern, even if player health is primary when providing medical care. It also cannot offer meaningful confidentiality protection given the doctor's obligations to advise the club about players’ health statuses (although our recommendations do not cloak player medical information in absolute confidentiality, for reasons discussed below). To the extent that the CBA provision is intended to provide players with unconflicted health care, it falls short because it does not resolve the foundational structural and institutional pressures club doctors face, whether implicitly or explicitly; the same problem plagues existing ethical and legal standards. So long as the club doctor is chosen, paid, and reviewed by the club to both care for players and advise the club, the doctor will have, at a minimum, tacit pressures to account for the club's best interests.^68^

Finding a solution to these problems is not easy. Many commentators before us have recognized the problems at hand, discussing conflicts of interest and pressure from the club on club medical staff, decisions about when a player can return to play, and player autonomy.^69^ Some have also recommended solutions. For example, in a 1984 article, Thomas Murray, now president emeritus of The Hastings Center, proposed four possible solutions for correcting conflicts of interest in sports medicine: clarifying the nature of the relationship at the outset, insisting on club doctors’ professional autonomy over the medical aspect of decisions, insulating the club doctor “structurally from illegitimate pressures,” and professionalizing sports medicine.^70^ We agree that the first two proposals would help,^71^ but we do not believe that they solve the structural conflict of interest that is at the root of the problem. The fourth proposal has seemingly largely come to fruition but without resolving the problem. The third proposal is promising but needs to be developed in more detail in order to be put into practice.^72^ It is, in our view, helpful foundational work.

There is a spectrum of possible approaches for changing the structure of the interactions between club doctors, players, and clubs. Each approach has its own benefits and deficiencies. Here are five possibilities, several of which could be combined or further dissected.

***A. Maintain the status quo but with increased reliance on personal and second‐opinion doctors.*** Players increasingly frequently obtain second opinions to compare against those provided by the club doctor,^73^ and they also rely ever more heavily on their own personal doctors for care. Nevertheless, as we discuss more fully in the longer “Protecting and Promoting the Health of NFL Players,” our interviews with players and contract advisors indicate that for some players, seeking care from a personal doctor is a burdensome process that they are reluctant to undertake. It is far easier for players to receive health care at the club facility, where they are already spending a considerable amount of their time, than to seek out a personal doctor whose office is off the premises and who may have a less robust understanding of a player's professional and physical challenges. The trouble of seeing a personal doctor is exacerbated by how much players travel and move during, after, and between seasons. Consequently, many players—particularly younger players—continue to rely solely on the medical opinion of and care provided by the club doctor. It is thus uncertain how practical recommending this approach is. Moreover, it does not resolve the fact that club doctors would remain in a conflicted position.
***B. Maintain the status quo but without the execution of confidentiality waivers.*** As we discuss above, players as a matter of course execute collectively bargained waivers that permit the club medical staff to disclose their health information to the club, stripping players of certain protections provided for in relevant laws and ethical codes concerning confidentiality. The players could refuse to execute these waivers and effectively preclude the clubs from knowing the specifics of a player's medical condition. However, it is unrealistic to expect players who are constantly under threat of having their contracts terminated to risk displeasing the club's management by taking this stand on their own; they would have to do so collectively, in a way supported by the NFLPA. More importantly, however, as we also discuss above, employers are arguably entitled to at least some information about an employee's work‐related health, and the club would still likely be entitled to know at least whether the player was fit to play—and to know that, the club may actually need access to quite a wide range of medical information. Thus, the players would gain little by refusing to sign the waiver, and the institutional and financial pressures concerning medical care provided by the club doctor would remain.
***C. Pay club doctors from a fund to which the NFL and the NFLPA jointly contribute.*** The core problem is that club doctors are hired, paid, and reviewed by the clubs although their services include treating players, whose interests may diverge from the clubs’. A structure in which the club doctor is paid equally by the NFL and NFLPA has the potential to remove some of the implicit structural pressures that the club doctor might feel to act in the club's best interests and marginally improve the trust of players. However, so long as the club doctor is chosen and reviewed by the club and is retained to provide services simultaneously to players and clubs, the doctor may still be under pressure to compromise the player's best interests in favor of the club's.
***D. Choose club doctors and subject them to review and termination through a committee of medical experts selected equally by the NFL and the NFLPA.*** 74 Another way to avoid the core problem is to incorporate the players into the hiring, review, and termination process for club doctors equally with the clubs themselves. For example, the NFL and NFLPA could each select three members of a committee, which could select a seventh neutral person as chair, and the committee could be made responsible for the selection, review, and potential replacement of the club physicians for each of the thirty‐two clubs. Additionally, this committee could be responsible for determining the doctor's compensation, taking into account the proposed rates by the doctors interested in the position and market rates in the club's city. The doctor's compensation could still be paid by the club, but the doctor could be subject to periodic review (perhaps once during the season and again after the season) in which the interested parties would have an opportunity to weigh in on the doctor's performance. The committee could also gather data on the performance of club doctors with the potential to enable the identification of “outliers” and take corrective action. If the committee determined that the doctor's performance was unsatisfactory, taking into consideration all of the parties’ needs, it should then also have the ability to terminate the doctor.
  This kind of solution would reduce the pressure some club doctors may feel to please the club in their treatment decisions and information disclosure, since their terms of employment would no longer be controlled by only one of the relevant parties. Importantly, it might also change players’ perceptions and promote trust. Adding another party might help resolve the conflict of interest we have identified. However, it would remain the case even under this approach that club doctors would be responsible to provide services to both players and clubs—and that alone could create conflicting obligations.
***E. Bifurcate doctors’ responsibilities between players and clubs.*** Finally, then, we should contemplate designating one doctor whose sole responsibility is to provide care to the players (call that person the “Players’ Doctor”) and another whose sole responsibility is to evaluate the player's fitness to play and advise the club accordingly (the “Club Evaluation Doctor”). This solution would avoid the dual‐loyalty problem by creating two completely separate medical roles, each with a single loyalty and a distinct set of responsibilities. Such a split has the potential to ensure that the player is receiving unconflicted medical care at all times while still allowing the club to receive the guidance it needs. In order for the Club Evaluation Doctor to be able to perform his or her job, however, he or she would need substantial access to the player and the player's medical information.

This proposal could help ensure that players receive care from a doctor who has only their best interests in mind, and whom they can trust to have only their best interests in mind. However, if the Players’ Doctor were still being selected exclusively by the club, a conflict of interest would remain. Additionally, the Club Evaluation Doctor may have a diminished capacity to provide an opinion as to whether the player is fit to play if he or she is not also treating the player personally, with all of the knowledge and understanding that the treatment relationship entails.

## Our Recommendation

Any of these approaches would be an improvement over the current situation. Each also has deficiencies, however. Our recommendation is to combine the last two. To address the inherent structural conflict of interest the club medical staff faces in the current arrangement and ensure that players receive medical care that is as free from conflict as possible, we recommend a division of responsibilities between two distinct groups of medical professionals. Player care and treatment should be provided by one set of medical professionals (the Players’ Medical Staff), appointed by a joint committee with representation from both the NFL and NFLPA, and evaluation of players for business purposes should be done by separate medical personnel (the Club Evaluation Doctor).

Here is how our recommendation would work. As discussed earlier, the CBA requires clubs to retain several different types of doctors. Currently, the use of these doctors and their opinions are largely filtered through the head club doctor, who visits the club's practices a few times a week, directs the athletic trainers, and otherwise generally leads the medical staff. This structure and process would largely remain, but with two important distinctions. First, doctors and the other medical staff for all of the clubs could be paid by the club but would be chosen, reviewed, and have their compensation determined by the committee of medical experts jointly selected by the NFL and NFLPA. Second, they would have as their principal obligation the treatment of players in accordance with prevailing and customary medical ethics standards and laws. For shorthand, we refer to the head doctor in this new role as the “Head Players’ Doctor,” and we refer to the collection of other doctors (and medical personnel mentioned earlier) as the “Players’ Medical Staff.”

The Head Players’ Doctor would effectively replace the individual currently known as the club doctor. In many respects, the daily responsibilities of the doctors and athletic trainers would not change under our proposed system. The key change, though, is that they would now work for the players rather than for the clubs. The Head Players’ Doctor would be at practices and games for the treatment of players and would be responsible for directing the work of the athletic trainers (who are also part of the Players’ Medical Staff). The Head Players’ Doctor and the entire Players’ Medical Staff would provide care and treatment to the players without any communications with or consideration given to the club outside of a *Player Health Report* that we discuss below. Moreover, the Head Players’ Doctor (with input from the player) would control the player's level of participation in practices and games. Again, even though the Head Players’ Doctor would still be paid by the club, he or she would be selected, reviewed, and, potentially, terminated by the medical committee, thus avoiding a key source of conflict. Such a review should include a determination of whether the Head Players’ Doctor has abided by all relevant legal and ethical obligations on top of an evaluation of his or her medical expertise.

The value of this approach is demonstrated by the unaffiliated neurotrauma consultant who currently exists as part of the NFL's concussion protocol. As discussed above, each club is assigned a neurotrauma consultant who is not affiliated with any club to help evaluate players for concussions during the game. In adopting this approach, the NFL and NFLPA have recognized and endorsed the importance of a player's receiving health care free from actual or potential conflicts of interest. It is our view that player health care should be free of conflicts of interest at all times, not only during examination for a possible concussion. In effect, our recommendation employs a structure that is already in place, merely extending it from treatment by the unaffiliated neurotrauma consultant to all medical encounters.

### The player health report

Under our recommendation, the club would be entitled to a regular written report from the Players’ Medical Staff about the status of any player currently receiving medical treatment. We call this the “Player Health Report.” Like many employers, clubs have a legitimate business interest (and indeed in many circumstances a legal right) to know about their employees’ health insofar as it affects their ability to perform the essential functions of their jobs. The Player Health Report would serve this purpose by briefly describing the player's condition, the player's permissible level of participation in practice and other club activities, the player's current status for the next game (out, doubtful, questionable, or probable), any limitations on the player's potential participation in the next game, and an estimation of when the player will be able to return to full participation in practice and games. The Player Health Report would be a summary form written for the lay coaches and club officials, as opposed to a detailed medical document. Generally speaking, we propose that the Player Health Report be provided to the club before and after each practice and game. Additionally, the club would be entitled to a Player Health Report on days where there is no practice or game if a player has received medical care or testing. The Player Health Reports should simultaneously be made available to players themselves, perhaps through their electronic medical records. The Players’ Medical Staff should complete the Player Health Report in a good‐faith effort to permit the club to be properly prepared for its next game.^75^

Generating the Player Health Report would be substantially similar to some of the current duties and requirements of club doctors. Club doctors and athletic trainers regularly update the club on player health status, and they are also required to advise a player in writing of any information that the club doctor provides to the club concerning the player's condition that “significantly affects the player's performance or health.”^76^ That player notification requirement would stand.

The important difference is that the Players’ Medical Staff's determination as to the player's status would now control the player's level of participation in any practice or game. If the Players’ Medical Staff declares via the Player Health Report that the player cannot play, then the club must accept that decision. If the club deviates from the limitations set forth in the Player Health Report, the club should be subject to substantial fines or other discipline under the CBA. The club, of course, would retain the right to not play the player for any number of reasons, including injury or skill level.

If a doctor hired by the club for the purposes of advising the club needs clarification from the Head Players’ Doctor concerning a player's status, then such communication should be permitted, as determined to be reasonably necessary by the Head Players’ Doctor. While it is expected that the players’ athletic trainers would help create the Player Health Report, communications between the Club Evaluation Doctor and the Players’ Medical Staff concerning player health should occur only with the Head Players’ Doctor. Beyond these minimal levels of communication, there should be no need for the Players’ Medical Staff (doctors and athletic trainers) to communicate with any club employee, including a coach or general manager. The goal of minimizing and formalizing the communication in this way is to minimize the club's ability to influence the medical care provided to the player, including subtle forms of influence such as through workplace conversations. We say “minimize” because our recommendation does still allow for some communications between the Players’ Medical Staff and the club. We think that this reduced level of communication is necessary and appropriate to protect player health, but we acknowledge that the existence of any such communications may cause a player to be less forthcoming to the medical staff, even if designated as the Players’ Medical Staff as we recommend.

In creating the Player Health Report, it is important that the Head Players’ Doctor take into consideration the player's desires rather than strictly clinical criteria. Like all patients, players are entitled to the right to make choices concerning their health care. Thus, if a player who is fully informed of the risks wishes to play through an injury, the Head Players’ Doctor should take that into consideration in completing the Player Health Report and deciding whether the player can play. However, players who have suffered concussions or other injuries that might affect the player's cognition at the time of decision‐making should be given significantly less deference.^77^

If the Head Players’ Doctor declares that a player cannot play but the player nonetheless wants to do so, then the player should be able to receive a second opinion. The logistics of when and how the player obtains the second opinion would need to be well coordinated; it would likely have to be a local doctor or practice group prepared to handle these situations for the players on short notice. If the second‐opinion doctor says the player can play, then the player should be allowed to make the decision about his status. Since players could end up shopping for doctors who will clear them to play, we recommend that the medical committee create a list of well‐qualified and approved second‐opinion doctors for the players to consult. This compromise also helps resolve concerns that the Head Players’ Doctor for one club might be overly conservative as compared to Head Players’ Doctors for other clubs. Nevertheless, during in‐game situations, the Head Players’ Doctor would retain substantial control over the player's participation, as is currently the case.

### The club's access to player medical records

The Player Health Report is distinct from the player's medical records. The Player Health Report is a limited view of the player's current health and provides information about the player's immediate or near‐immediate availability to the club. A player's complete medical record provides a fuller picture of the player's health and would provide additional information needed for assessing a player's long‐term health, as well as a separate check on the assessment provided in the Player Health Report.

Under our recommendation, in addition to the Player Health Report, the club would be entitled to the players’ medical records, as is the case under the status quo. We reiterate the clubs’ legitimate business need for a clear understanding of player health issues. Clubs would obviously and rightfully be interested in understanding a player's medical condition in both the short‐ and long‐term. While some might believe that clubs should be entitled only to those medical records that are specifically relevant to football, in reality, this line cannot easily be drawn. Clubs might believe that most of a player's medical issues, concerning both physical and mental health, are relevant to the player's status with the club. That said, as we discuss in a forthcoming article, there may be important legal restrictions on the request for and use of some of that information by an employer, including constraints imposed by the Americans with Disabilities Act and the Genetic Information Nondiscrimination Act.^78^

Providing clubs access to players’ medical records creates some complexities concerning athletic trainers. These trainers are the principal providers of medical care to players under the control of club doctors and also are generally responsible for completing the players’ medical records. We think athletic trainers could retain these roles, but we recommend that they, like the Head Players’ Doctor and Players’ Medical Staff, be chosen and reviewed by the medical committee and that their principal obligations be to treat the players in accordance with prevailing and customary legal and ethical standards. The athletic trainers would probably assist the Head Players’ Doctor in creating the Player Health Report, but like the Head Players’ Doctor, they should have minimal, if any, other interaction with the coaches or other club officials.

### Club evaluation doctors

Under this new approach, clubs would be free to retain doctors and other medical professionals, as needed, who work solely for the clubs for the purposes of examining players and advising the club accordingly. These doctors, whom we call “Club Evaluation Doctors,” could perform the pre‐employment examinations at the Combine, during the course of free agency, and also examine players during the season. However, they would not treat the players in any way. The Standard Player Contract's requirement that players make themselves available for an examination by the club doctor upon request would largely remain. Additionally, the Club Evaluation Doctor would have the opportunity to review the players’ medical records at any time and communicate with the Head Players’ Doctor about the Player Health Report, if clarification were needed and appropriate. As discussed below, the Player Health Report should substantially minimize the need for duplicative medical examinations. This arrangement would thus permit a Club Evaluation Doctor to provide an opinion as to a player's short‐ and long‐term usefulness to the club, without relying on the Players’ Medical Staff's opinion.

### Support from ethical principles

This recommendation brings interactions between club medical personnel and players into better alignment with the ethical principles that we suggest should govern. It shows respect to players as autonomous individuals, with long‐term interests beyond their playing days, by providing them with medical professionals who are devoted to their well‐being rather than forced to serve two parties and find a way to walk a line between duties to the player and duties to the club. It better balances health primacy and empowered autonomy, by providing players access to medical staff focused on the players’ best interests. It promotes transparency by making clear to players when a doctor is acting as their uncompromised partner in health and removing the possibility that the doctor may be advancing the interests of the club. Most importantly, it helps manage conflicts of interest, although it does not completely eliminate them.

Splitting responsibilities and roles between Players’ Medical Staff and the Club Evaluation Doctor could also promote collaboration and engagement between the clubs and the players, rather than placing them at odds with each other, with the doctors caught in the middle. It would allow clubs and doctors to promote player health without sacrificing the club's valid interests in evaluating players. At the same time, players would now have the opportunity to be more engaged in their own health outcomes. Our recommendation also expands opportunities for the NFL and NFLPA (as the players’ representative) to come together in evaluating the qualifications and performance of the medical personnel who treat players.

Finally, the recommendation reflects the principle of justice. It recognizes that players are not homogeneous. Under the current system, some players have access to excellent health care outside the club and are sophisticated about how the information they provide to club doctors may be used against them in their evaluation, while others (particularly younger players) may lack that access and not have acquired that sophistication. By providing every player with a doctor committed solely to his health and freed from the impediment of a structural conflict of interest, our proposed arrangement would attempt to even the playing field.

### Possible objections to our recommendation

We acknowledge that there may be concerns with our recommendation. As we evaluated the options, we sought the opinions of others, including several medical and sports medicine professionals. Indeed, some of the peer reviewers of our main report, “Protecting and Promoting the Health of NFL Players,” expressed concern about overly limiting communication between player medical staff and the club, resulting in our decision to broaden the scope and frequency of permissible communications compared to our original position. By contrast, some viewed the extent of communication that we allow as too substantial. Outside of the context of professional sports, however, personal doctors do occasionally communicate with a patient's employer in ways sanctioned by that patient (for example, providing information to justify sick leave). Thus, we believe that this final recommendation describes the best way to serve the goal of providing players with health care they can trust, from providers who are as free from conflicts of interest as possible, while acknowledging the business realities facing clubs. We recognize that it might need further adjustment as it is implemented and that making the transition to it might be challenging, but we think that it is feasible.

From the players’ perspective, there are at least five possible objections to our recommendation:

First, some may question why we have not advocated for a complete bifurcation of roles, where there is one set of doctors that provides players with care and has no relationship or communication with the club whatsoever and another set that provides advisory services to the club, including performing medical examinations of players. In other words, why not extend our recommendation to prohibit all communication, including the Player Health Report, between the Head Players’ Doctor and the Club Evaluation Doctor?

The answer is that we believe several considerations make such a prohibition impractical. First, prohibiting all communication between the doctor caring for the player and the club would require the club to perform its own independent assessment of the player for every condition, which would probably subject many players to duplicative examinations. Our provision for a Player Health Report would minimize this problem by allowing some flow of information and communication. Second, as we discussed above, we believe clubs have a legitimate right to a player's health information and status insofar as they can affect his ability to play. Third, to the extent that clubs would receive information about a player's health from the player himself, completely bifurcating roles and cutting off communication imposes an unnecessary burden on the players and creates a risk of miscommunication and lost information. Additionally, there are questions about whether players would adequately track and seek reimbursement for out‐of‐pocket health care expenses.

Second, some may object that our recommendation does not completely eliminate the confidentiality concerns that exist under the current model because the club would still receive medical information concerning players. This objection is correct, and it may cause players to refrain from full disclosure of their ailments to the Players’ Medical Staff. However, despite this confidentiality concern, we anticipate that having a medical staff fully devoted to the players’ interests will facilitate a player's trust that the care he is receiving supports and promotes only his—and not the club's—best interests. Again, we think that passing at least some medical information to the club is a necessary business reality.

Third, some might wonder whether it is preferable to have players select the members of the medical committee directly rather than via the NFLPA. Such an approach would give the players more direct input into their medical care. However, in addition to the fact that the NFLPA is the players’ representative, it has experience in this type of neutral selection process. Many such processes are already called for in the CBA (as for the System Arbitrator, Noninjury Grievance Arbitrator, and Benefits Arbitrator).^79^ Additionally, the NFLPA has more time to devote to the selection process and to any subsequent issues than players would. Finally, the benefit of developing institutional knowledge over time would be challenging for a player to attain during his career.

Fourth, some might question why the NFL would be allowed any role in the selection of Players’ Medical Staff, even if part of a balanced medical committee. The reason, again, is that clubs have legitimate business‐related interests in the health of their players. While these interests likely sometimes conflict with a player's interests, there is also an alignment of interests: one would generally expect that clubs have an interest in their players’ receiving the best possible health care—if for no other reason than to protect the clubs’ investment in their players. Indeed, clubs invest considerable sums in players and the business of the NFL. Moreover, clubs and the NFL already have substantial knowledge about which doctors are well qualified to provide health care to NFL players. Consequently, it is appropriate that the NFL be involved as a voice, but not a controlling interest, in the composition of the medical committee.

Fifth, some might disagree with the structure of our recommendation insofar as the Head Players’ Doctor, Players’ Medical Staff, and athletic trainers would all still be paid by the club. Some might believe that receiving a paycheck from the club could cause the Players’ Medical Staff to (at least subconsciously) favor the club's interests. In the abstract, there is some merit to this point, based on what we know about subtle conflicts of interest.^80^ However, the conflict here is not really the source of payment but, rather, the locus of control over hiring and firing. Having the medical committee hire and review the doctors and athletic trainers and determine their level of compensation is enough to manage the structural conflict of interest; it ensures that the Head Players’ Doctor has every reason to be concerned only about the players’ interests. Consequently, it does not seem necessary to introduce the logistical complexity of having a third party pay the Players’ Medical Staff.

From the clubs, at least three other objections might arise. First, they might object to having to retain their own doctors (in some capacity) and perhaps additional specialists. Currently, clubs typically pay for two levels of care: the primary care by the club doctor and a second opinion obtained by the player.^81^ Our proposed structure creates a potential third layer of medical examination, that of the Club Evaluation Doctor. Nevertheless, we disagree with this objection, for several reasons. First and foremost, our proposed structure is essential for players to receive minimally conflicted health care. Second, with a Head Players’ Doctor who is entirely devoted to the player's interests, players should have an increased level of trust in their primary level of care, which can decrease the need (and cost) of second opinions (although we recognize we may not conclusively know the effect on the bottom line until after the system is implemented). Third, clubs would also benefit from our recommended arrangement because they would have a Club Evaluation Doctor entirely devoted to the club's interests. Finally, at least under the current CBA, some of the costs of medical care, including physical examination costs, are paid for at least partially out of the players’ share of revenue.^82^ Any additional costs for player health care only decrease the amount of money available to players in salary.^83^

Second, clubs might object that players already have access to their own doctors, second‐opinion doctors, and the surgeon of their choice. This is correct, but the level of access to these alternative doctors as compared to the current club doctors is dramatically different. Given the time demands placed on them by the club, travel schedules, and movement between clubs, it is far easier (and more realistic) for players to receive medical care at the club facility from the club doctor now—or the Players’ Medical Staff under our proposed arrangement. Additionally, players’ personal doctors and second‐opinion doctors are not there on the sidelines of games when important medical decisions are often made. Finally, under our recommendation, the Head Players’ Doctor would have control over whether a player plays, which is not an authority that a player's personal or second‐opinion doctor could have.

Third, clubs might believe that coaches and club executives need to be able to speak directly to the Players’ Medical Staff to be able to properly understand a player's condition and limitations. We recognize this concern and acknowledge that the proposed Player Health Report is a substantial departure from existing practices, whereby athletic trainers communicate regularly with the coaches and general manager. Consequently, we understand that there will be resistance to change and legitimate logistical challenges in transitioning to a new set of protocols. Nevertheless, we believe that clubs could adjust to a new structure. Ultimately, with the help of existing NFL club doctors and athletic trainers, the proposed Player Health Report could be crafted and implemented so that it provides clubs with the information they need to evaluate a player's fitness to play. Additionally, to the extent that clubs believe they need additional clarification, the new Club Evaluation Doctor could communicate with the Head Players’ Doctor or athletic trainers or could examine a player directly, as appropriate.^84^

Outside of the player‐ and club‐centric perspectives, there might be other concerns with our recommended approach. The Head Players’ Doctor might be a fan of the club or might idolize the players in some way, and either attachment could affect the care and advice provided to the player. This is an issue the medical committee would have to evaluate. Additionally, players can always hide their conditions in efforts to convince the Head Players’ Doctor to permit them to play. Nevertheless, we believe that this recommendation could substantially resolve the major concern about the current club doctor arrangement—the problem of dual loyalty and structural conflict of interest—by providing players with a medical staff that has only the interests of the players in mind. The Head Players’ Doctor would be almost entirely separated from the club and the pressures implicit in being employed by the club while being held accountable to a neutral medical committee. At the same time, our recommendation does not interfere with the clubs’ legitimate interests. For these reasons, we believe that this recommendation is critical to improving player health and among the most important set forth in our report “Protecting and Promoting the Health of NFL Players.” Accordingly, it and all of its intricacies should be set forth in the CBA.

## From the Club Medical Staff to the Microenvironment Affecting Player Health

In this article, we have homed in on the responsibilities of and recommendations about one group of stakeholders with an obvious and essential role in protecting and promoting player health. But the medical staff is far from the only stakeholder capable of making a positive difference. Indeed, the more detailed and comprehensive “Protecting and Promoting the Health of NFL Players” from which this discussion of club doctors is derived aims to answer some broader questions: Who is responsible for the health of NFL players, why, and what can be done to promote player health? To date, there has been no broad analysis of the various stakeholders that may influence player health, nor any systematic analysis of their existing or appropriate legal and ethical obligations. However, this sort of undertaking is essential to uncovering areas in need of improvement and making clear that the responsibility for player health falls on many interconnected stakeholders that must work together to protect and support players.

In addition to club doctors, who are these stakeholders? Players are, of course, the center of the universe in any analysis of player health. After all, it is they who make many of the key decisions that can protect and promote their health, or fail to do so. But it is often not as simple as saying, “If you're hurt, don't play,” or, “If you're worried about the risks, find something else to do.” The sorts of constraints faced by players include not only the kinds of limitations we all face as imperfect decision‐makers—for example, biases that lead us to believe that statistical predictions about unpleasant outcomes will not apply to us or to give more weight to our current needs and desires than to those of our future selves—but also financial, legal, and social structures that may constrain or shape available decisions. Players are offered lucrative but tenuous contracts; they may feel pressure from teammates, coaches, fans and others; and they often love the game, regardless of physical limitations or consequences. None of this is to suggest that professional players are not competent moral agents, making voluntary decisions to play football. They certainly are. Still, improving the background circumstances that influence their decisions—and that differ for each player—can be a good idea. Thus, while we recognize that players surely bear responsibility for their own health, in many cases players cannot protect and promote their health entirely on their own, nor may they treat health as their unyielding primary goal.

Although the competitive nature of the game and the limited available roster spots are inherent features that will not change, players need a structure that helps them make decisions that advance their own interests, as they define those interests. They need accurate information (yet this has not always been available, and significant uncertainties in available scientific data remain),^85^ unconflicted practitioners and advisors, social support and safety nets when they make choices that turn out poorly, easily accessible opportunities to prepare for life after football, and a cultural shift toward greater respect for and understanding of players who take steps to protect their health. On top of all this, occupational safety and health laws in the United States prevent individuals from consenting to any workplace risk they may be willing to accept.^86^ Instead, employers are required to take various steps to protect against such risks. As discussed in further detail in “Protecting and Promoting the Health of NFL Players,” it is clear from both legal and ethical perspectives that respect for individual autonomy in the face of even substantial risks must be paired with reasonable efforts by the NFL and others to abate risk exposure.

**Figure 1:**
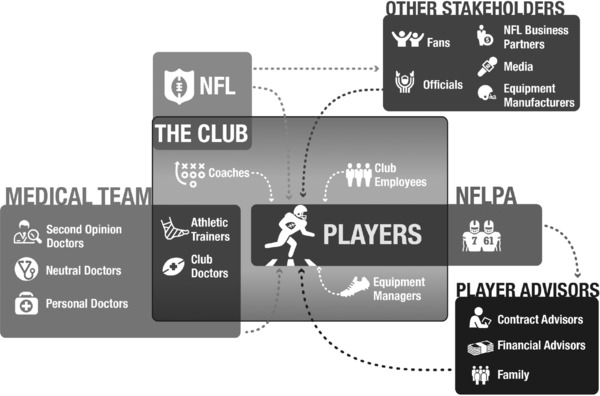
Stakeholders in Players’ Health

Thus, while recognizing a critically important role for players, we also view a variety of additional stakeholders who can be key influences, for good or for bad, on player health. In our view, the key stakeholders are the players themselves, the club doctors, athletic trainers, second‐opinion doctors, neutral doctors, personal doctors, the NFL, the NFLPA, the various NFL clubs, coaches, other club employees, equipment managers, contract advisors, players’ financial advisors, family members, officials, equipment manufacturers, the media, fans, and NFL business partners (see figure 1).

This list of stakeholders incorporates three subsets: those who directly affect player health, for example, as employers or caregivers; those who reap substantial financial benefits from players’ work; and those who have some capacity to influence player health. The result is a complex web that must be examined to improve player health. Some aspects of this larger web are especially important. Medical sponsorships (in which doctors advertise their relationships with the club) and prescription and painkilling drug practices warrant close analysis, for example.

These issues are discussed fully in “Protecting and Promoting the Health of NFL Players.” Here, we have focused on the structure in which club doctors provide care. This structure—which is flawed even in the absence of ethical lapses by any individual club doctor—may substantially contribute to player health concerns. The CBA, existing ethical standards, and current laws do not adequately address the structural conflicts because they do nothing to address the problem that club doctors wear two hats, providing services simultaneously to players and clubs. Our recommendation to separate these two roles currently played by club doctors and to establish a new group of medical professionals dedicated exclusively to players can resolve the structural problems in a major way while being responsive to practical realities.
